# Rb substantially compensates for the double loss of p130 and p107 in adult but not embryonic neural stem cell lineages

**DOI:** 10.1038/s41419-025-07815-6

**Published:** 2025-07-10

**Authors:** Reem Swaidan, Ahmad Daher, Anthony T. Bejjani, Yara E. El Atie, Yasmina Chehab, Razan Bou Hamdan, Renaud Vandenbosch, Ruth S. Slack, Saad Omais, Noël Ghanem

**Affiliations:** 1 Department of Biology, Faculty of Arts and Sciences, American University of Beirut, Beirut, Lebanon; 2 Laboratory of Developmental Neurobiology, GIGA Institute, University of Liège, Liège, Belgium; 3 Division of Histology, Department of Biomedical and Preclinical sciences, University of Liège, Liège, Belgium; 4 Department of Cellular and Molecular Medicine, University of Ottawa, Ottawa, ON Canada; 5 Department of Biological Sciences, School of Arts and Sciences, Lebanese American University, Beirut, Lebanon

**Keywords:** Adult neurogenesis, Developmental neurogenesis

## Abstract

The Retinoblastoma (Rb) family of pocket proteins (p107, Rb, and p130) controls all aspects of neurogenesis from stem cell activation to long-term neuronal survival in the brain. Previous studies have reported non-overlapping, often complementary, roles for these cell cycle regulators with possibility for functional compensation. Yet the extent to which each protein might compensate for other family members and whether synergistic effects exist during neural stem cell (NSC) lineage development remain unclear. Fong et al. recently revealed that a triple knock-out (TKO) of all pocket proteins results in a transcriptomic switch from NSC quiescence to activation, followed by niche depletion in the adult hippocampus. Here, we investigated whether pocket proteins are equally critical in NSC fate regulation in the adult subventricular zone (aSVZ) and during embryogenesis. We report that TKO of these proteins results in NSC activation coupled to ectopic progenitor proliferation and massive apoptosis, leading to niche depletion and premature loss of neurogenesis inside the olfactory bulb (OB). Notably, a p107–p130 double knockout carrying a single wild-type Rb allele (DKO) substantially rescues the above defects and maintains adult neurogenesis. In comparison, TKO embryos display severe disruptions in all stages of neurogenesis at E14.5, leading to embryonic lethality. Similar defects are detected when any five out of the six alleles of pocket proteins are lost, with only partial rescue of the proliferation defects observed in DKO embryos. The above TKO phenotypes are partially mediated by opposed deregulations in the Notch-Hes signaling pathway in the embryonic versus the adult brain. Such deregulation is linked to opposite changes in E2F3a and E2F3b embryonic gene expressions. Our data identifies Rb as a critical pocket protein in the control and maintenance of adult OB neurogenesis, and uncovers interchangeable, dose-dependent roles for pocket proteins in the control of neuronal differentiation and survival during development.

## Introduction

Rb, p107, and p130 constitute the Rb family of “pocket proteins,” where they function to regulate cell cycle at the G/S1 checkpoint in all cell types [1]. While initially studied for their role in cancer development, these proteins were also found to regulate all aspects of neurogenesis in the embryonic and adult brain, from stem cell activation to long-term neuronal survival [2–4]. Several reports have suggested non-overlapping (often complementary) requirements for pocket proteins, as well as a possibility for functional compensation. Yet the extent to which each pocket protein might compensate for other family members remains unclear in many contexts [5]. Unraveling the interactions of these proteins in each tissue will help identify which of the three is most indispensable in any given species, cell type, and/or developmental stage.

Perhaps the most obvious example of compensatory effects among pocket proteins occurs during retinoblastoma formation in the murine retina, where upregulated p107 levels prevent tumorigenesis following loss of Rb. In the human retina, however, a failure to increase p107 levels in response to Rb loss causes retinoblastoma [6, 7]. In addition to species-specific feature of compensation, tissue-specific constraints exist as well. Unlike the retina, Rb inactivation alone can still induce osteosarcomas, thyroid, and pituitary cancers in mice [8]. Functional redundancy might also become more prevalent in adult/aged tissues compared to embryogenesis, thereby possibly explaining the increased dispensability of Rb in regulating neurogenesis with age [9, 10].

During neurogenesis, p107 acts similarly in embryonic and adult neural lineages to control stem cell activation and neuronal commitment [11, 12]. Rb, in contrast, regulates progenitors’ proliferation, neuroblast migration, and cortical development [13–15] and continues to control progenitors' proliferation in the young aSVZ as well as long-term neuronal survival inside the OB [16]. p130 remains the least studied of all three proteins, where it is believed to maintain survival of postmitotic mature neurons [17]. Few studies have addressed the combined roles of pocket proteins in regulating neurogenesis. Svoboda et al. showed that Rb and p107 are required for radial migration and cortical lamination independent of their role in apoptosis, a requirement that does not hold for Rb alone [18]. Other reports showed that mature cortical neurons only undergo apoptosis if all pocket proteins are deleted following differentiation, but not before; this is due to a protective mechanism that involves Chk1/Atm pathway activation [19, 20]. Most recently, Fong et al. reported severe defects in stem cell activation, causing niche depletion in an inducible TKO of all pocket proteins in the adult hippocampus [21, 22]. Whether TKO mice display comparable neurogenic defects along the aSVZ-OB axis and whether compensatory effects are equally distributed between Rb, p107, and p130 in the regulation of neural stem cell lineages remains unknown.

Using Nestin-CreERT2-YFP inducible mouse lines, we investigated here potential compensatory and synergistic roles for Rb in the aSVZ of p107–p130 double knockout (DKO) mice carrying a single wild-type Rb allele in comparison with triple knockout (TKO) mice and triple heterozygous (THC) control mice. We also assessed similar mechanisms during embryogenesis. Our findings revealed severe neural lineage defects in adult TKO mice, leading to NSC depletion and loss of OB neurogenesis over time, a phenotype that is remarkably rescued in DKO mice. In comparison, TKO embryos displayed severe disruptions in all stages of cortical neurogenesis at E14.5, out of which only the proliferation defects can be rescued in DKO embryos. Interestingly, similar neuronal differentiation and survival defects are observed when any five out of the six alleles of pocket proteins are lost. We show that the above embryonic and adult TKO phenotypes are partially mediated by opposing deregulations in the Notch-Hes pathway, which are directly linked to an imbalance in E2F3a and E2F3b gene expressions during development.

## Materials and methods

### Animal maintenance and genotyping

All animal experiments and procedures were performed according to the standard protocols approved by the “Institutional Animal Care and Use Committee” (IAUAC) at the American University of Beirut. TKO mice were provided by Dr. Julien Sage [23] and crossed with Nestin-CreERT2 [24] and R26-stop-enhanced yellow fluorescent protein (YFP) mice [25]. Details about mice strains, breeding schemes, and genotyping were previously described [21]. Both females and males were used in all experiments.

### Tamoxifen treatment

For Cre induction, 2-month-old mice were administered tamoxifen (Sigma-Aldrich T5648) at 180 mg/kg/day for 4 days by gavage or by intraperitoneal injection (IP; for the 8wpt timepoint only) (dissolved in 10% EtOH / 90% corn oil). Pregnant females received a single tamoxifen dose at E10.5.

### Tissue preparation and cryoprotection

Mice euthanasia and brain tissue cryopreservation and sectioning were performed as previously described [16].

### Immunohistochemistry and Western blot analysis

IHC was performed as previously described [16]. The primary antibodies used are: Hes1 (mouse, 1:20, DSHB PCRP-HES1-2E8-s) and Hes3 (mouse, 1:20, DSHB PCRP-HES3-1A10-s), vimentin (mouse, 1:20, DSHB 40E-C-S), and PH3 (rabbit, 1:1000, Millipore 06-570). Secondary antibodies were purchased from Jackson Immunoresearch. Refer to Fong et al. for a full description of all other primary antibodies used and for details about Western blot analysis [21].

### In situ hybridization

In situ hybridization was performed as previously described [26]. Antisense riboprobe (600 bp) for Hes5 was prepared from plasmid [27]. Notch2 (509 bp) and RBPJ (873 bp) transcript fragments were amplified from E13.5 cDNA library using the following primers: Notch2 Forward (5′ cacgctttgcagtgtcgagg 3′), Notch2 Reverse (5′ tggcagcgtcctggaatgtcac 3′), RBPJ Forward (5′ gcagctgaacttggaaggga 3′), and RBPJ Reverse (5′ cacagagcatgctctctccac 3′). A T7 promoter (5′ taatacgactcactataggg 3′) was added ahead of each reverse primer to synthesize the antisense riboprobes.

### Quantitative Real-Time PCR analysis

Total RNA was isolated from the embryonic forebrain at E14.5 using the RNeasy Plus Mini Kit (Qiagen #74134). cDNA synthesis was done using QuantiTect Reverse Transcription Kit (Qiagen # 205311). qRT-PCR assays were performed with the iTaq™ Universal SYBR Green Supermix (Bio-Rad # 1725121). Values obtained for E2f3/4 expression levels were normalized to the 18S level as an internal control. The following primers were used: E2F3 for. 5′ gaaagcccctccagaaacga 3′ and rev. 5′ gggtctgtgtgtttccgtct 3′, E2F3a for. 5′ ggcgagaaggagagacttgg 3′ and rev. 5′ tcatctctcgctcctgctct 3′, E2F3b for. 5′ tcggaaatgcccttacagca 3′ and rev. 5′ gcagctcttcctttgccctt 3′, and, E2F4 for. 5′ tgcagatgctttgctggaga 3′ and rev. 5′ ctagcagcacctcgataggc 3′.

### Imaging, cell counts, and statistics

Images were captured using the Leica DM6B microscope with UV light and digital camera. Image analyses were performed using the Leica Application Suite software (LAS X) and ImageJ. Brain sections were matched at medial level from all genotypes and time points, and cell counts were done on 3–4 non-consecutive sections per sample. Raw counts were normalized to the perimeter area in mm in the SVZ and RMS, and surface area in mm^2^ inside the adult OB and the embryonic DC. The experimenters were blinded to the genotypes during counting.

All statistical comparisons were performed using an independent two-tailed Student’s t-test using the SPSS 26 software. Differences were considered significant with a p-value of <0.05 (*), **p < 0.01, ***p < 0.001. All data is presented as the arithmetic mean, plus or minus the standard deviation (mean ± SD). For each experiment, a minimum of 3 animals per genotype were used. All graphs were generated with GraphPad Prism 8 software.

## Results

**Fig. 1:**
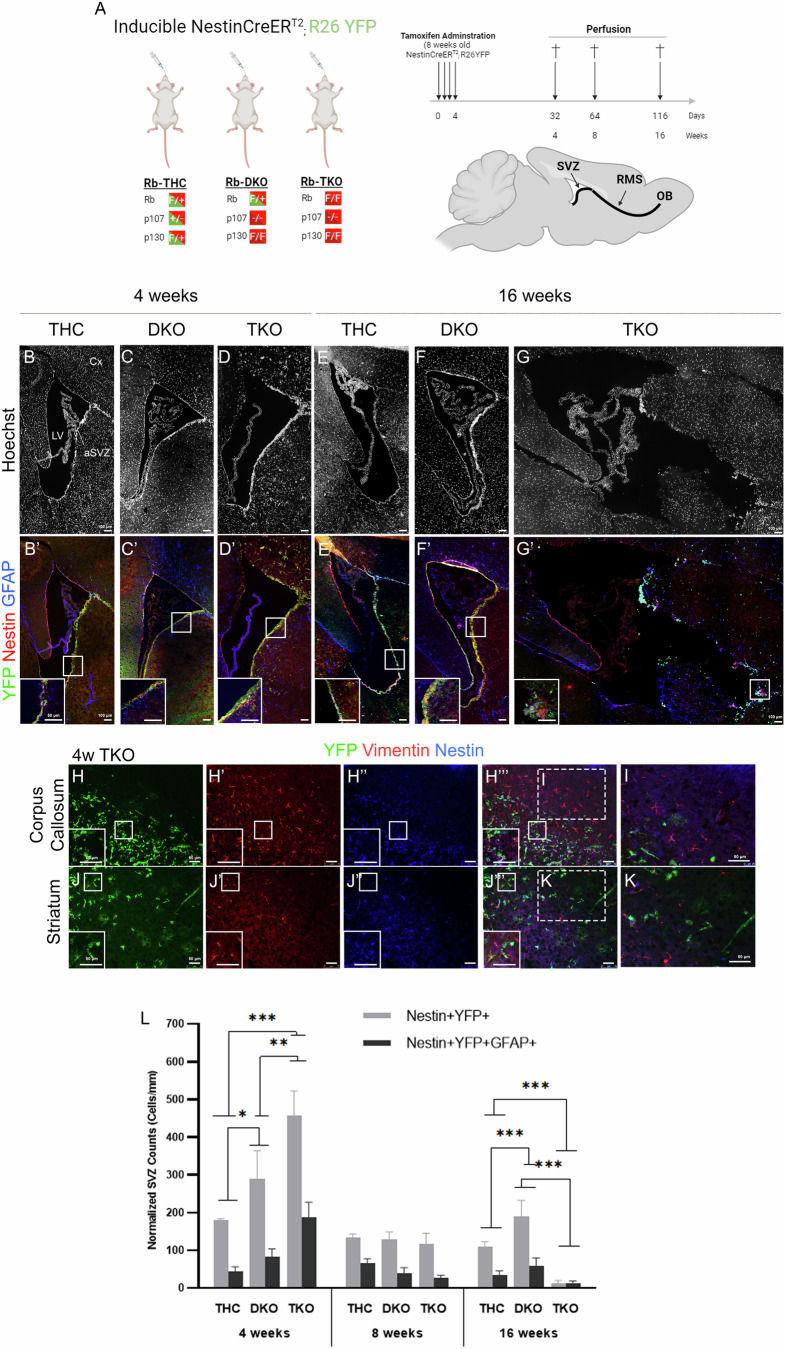
aSVZ-NSCs undergo fast activation and niche depletion over time in TKO but not DKO mice. **A** Experimental design illustrating tamoxifen treatment and collection periods of adult mice carrying inducible and compound deletion(s) in pocket proteins. Immunohistochemistry (IHC) on brain sagittal sections in the aSVZ at 4wpt and 16wpt in THC (**B**, **B’**, **E**, **E’**), DKO (**C**, **C’**, **F**, **F’**), and TKO (**D**, **D’**, **G**, **G’**) mice. Insets in (**B’**–**G’**) show higher magnification images of the boxed regions inside the aSVZ. Note the lateral ventricle’s enlargement and niche depletion in TKO brains at 16wpt. IHC in the corpus callosum (**H**, **I**) and striatum (**J**, **K**) at 4wpt showing vimentin-positive progeny with astrocyte morphology derived from aSVZ-NSCs, including some progenitors co-expressing YFP and/or Nestin. Insets in (**H**–**J”’**) are higher magnification images of the boxed regions. **I**–**K** Higher magnification images of dashed boxes in (**H”’**, **K”’**). **L** Quantification of cell counts in the aSVZ showing significant increase in (Nestin + YFP+) and (Nestin + YFP + GFAP+) cell populations in TKO and DKO compared with THC at 4wpt, followed by gradual decrease and stem cell pool depletion in TKO brains (but not DKO) by 16wpt. Cx cortex, LV lateral ventricle, aSVZ adult subventricular zone. Scale bars, (**B**–**G’**): 100 µm, (**H**–**K**): 50 µm. Error bars, mean ± SD. Unpaired 2-tailed Student’s t-test; *p < 0.05, **p < 0.01, ***p < 0.001. n = 3 biological replicates.

To investigate whether pocket proteins play synergistic roles in regulating SVZ-OB neurogenesis, we examined here the phenotypes of young adult mice carrying, in combination, double or triple deletions of Rb protein family. Specifically, p107-null mice carrying Rb and p130 double floxed alleles (p107^−/−^; Rb^flox/flox^; p130^flox/flox^) were mated with Nestin-CreER^T2^; Rosa26^YFP^ mice to generate triple knockout (p107^−/−^; Rb^f/f^; p130^f/f^) mice, referred to as (**TKO**) as well as triple heterozygous mice (p107^+/−^; Rb^+/f^; p130^+/f^) or (**THC**) that showed a similar phenotype to No Cre-THC and thus served as controls as previously reported [21]. Moreover, we generated double knockout mice (**DKO**) for p107 and p130 carrying one functional Rb allele (p107^−/−^; Rb^+/f^; p130^f/f^). Adult mice were treated with tamoxifen and sacrificed at either 4 weeks (**4wpt**), 8 weeks (**8wpt**), or 16 weeks (**16wpt**) post-treatment (Fig. 1A). Proof of Cre recombination and successful proteins’ deletions is shown in Supplementary Fig. 1A.

### TKO mice exhibit NSC activation and depletion in the aSVZ over time, a phenotype largely rescued by a single Rb wild-type allele

We first investigated the compound roles of pocket proteins on stem cell activation and progenitor proliferation by co-immunostaining for YFP, GFAP, and Nestin in THC, DKO, and TKO brains. Cre recombination was successful in the aSVZ; the percentages of [Nestin + YFP+] double-positive cells in the total Nestin population ranged between 92–97% at 4wpt, 87–93% at 8wpt, and 75–98% at 16wpt across all genotypes.

At 4wpt, our results showed a significant increase in the size of the [Nestin + YFP+] population per mm in the aSVZ in TKO and DKO compared to THC brains [ratio of (DKO/THC) = 1.6 and (TKO/THC) = 2.5]. At 16wpt, this increase was sustained in DKO brains (r = 1.7) but reached a critically low level in TKO brains (r = 0.12). Similarly, the ratios of activated NSCs [YFP + GFAP + Nestin+] were 1.93 and 4.4 in DKO and TKO brains compared to THC at 4wpt, respectively. Yet, at 16wpt, the latter population sharply declined in TKO brains (r = 0.37) but not DKO (r = 1.8) (Fig. 1B–G’, L). This increase in aNSCs pool is primarily due to loss of p107 that was shown to negatively regulate quiescent NSCs’ self-renewal [11]. Notably, many astrocyte-like cells were detected inside the striatum, the dorsal cortex, and the corpus callosum in TKO brains only. The majority of these cells stained positive for vimentin and some co-expressed YFP and/or Nestin, thus confirming their astrocytic lineage being derived from aSVZ-NSCs (Fig. 1D, D’, H–K). THC and No Cre-THC mice showed a similar phenotype at all three time points (Supplementary Fig. 1B).

This data shows that the combined loss of all pocket proteins causes a fast activation of NSCs, leading to pool depletion; a phenotype that can be rescued by a single functional Rb allele.

### Ectopic progenitor proliferation and ectopic neuroblast migration in TKO brains are prevented in DKO mice

**Fig. 2:**
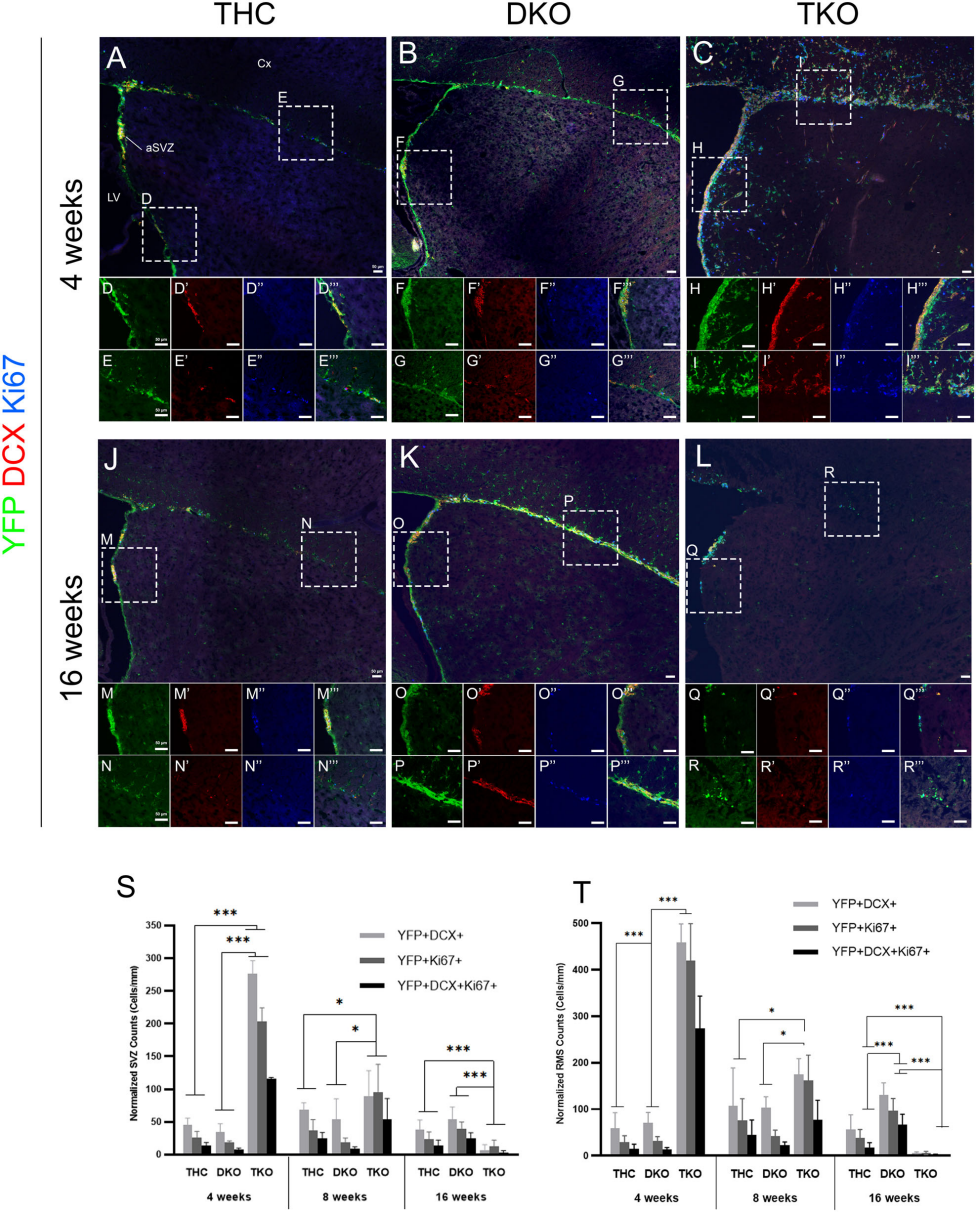
Ectopic progenitor proliferation and massive neuroblast migration in TKO brains are rescued in DKO mice. IHC on brain sagittal sections in THC (**A**–**E”’**, **J**–**N”’**), DKO (**B**–**G”’**, **K**–**P”’**), and TKO (**C**–**I”’**, **L**–**R”’**). Inset panels show higher magnification images of dashed boxes in the aSVZ and rRMS. Note the ectopic progenitor proliferation and neuroblast migration inside the dorsal cortex, corpus callosum, and striatum in TKO but not DKO mice at 4wpt compared with THC. Quantification of cell counts in the aSVZ (**S**) and rRMS (**T**). Compared with THC, TKO mice show a robust increase in (YFP + DCX+), (YFP + Ki67+) and (YFP + DCX + Ki67+) cell populations at 4wpt followed by gradual decrease, then loss of most dividing progenitors and migratory neuroblasts by 16wpt. DKO mice maintain a strong level of AN compared with THC at 16wpt. Scale bars, 50 µm. Error bars, mean ± SD. Unpaired 2-tailed Student’s t-test; *p < 0.05, **p < 0.01, ***p < 0.001. n = 3–4 biological replicates.

Next, we assessed cell proliferation and early neuronal differentiation by co-immunostaining for YFP, Ki67, and DCX. We did not score any difference between THC and No Cre-THC brains (Supplementary Fig. 1C, D). TKO brains showed excessive progenitor proliferation and massive neuronal differentiation compared with THC at 4wpt and 8wpt, but to a less extent. This is evidenced by ~4-fold increase in the thickness of the aSVZ and rostral RMS (rRMS) in TKO as estimated by the numbers of Hoechst-positive cells and [YFP + DCX+] neuroblasts, respectively (Figs. 1B–D and 2A–C, S, T). We thus detected a 7.1- and 5.9-fold increase in single Ki67+ cells and DCX+ cells at 4wpt, and, a 2.6- and 1.4-fold increase at 8wpt inside the aSVZ in TKO versus THC (Fig. 2A, C, D–D”’, H–H”’ and Supplementary Fig. 2A). [YFP+Ki67+] and [YFP + DCX+] cells were also significantly higher in TKO and ranged between 202-276 cells/mm as opposed to 25-46 cells/mm in THC at 4wpt. This was also true for [YFP + Ki67 + DCX+] cells (TKO:115 ± 2.2 vs THC: 13.4 ± 5.1) at 4wpt, and less so at 8wpt (Fig. 2A, C, S). TKO mice frequently displayed ventricular heterotopias along the ventral SVZ and lateral ventricles’ enlargement (Supplementary Fig. 2B). DKO brains did not show any of the above TKO defects.

In the rRMS, TKO mice showed a similar phenotype compared with the aSVZ. They displayed a 12.2- and 7.7-fold increase in single Ki67+ cells and DCX+ cells at 4wpt, and, a 2.1 and 1.7-fold at 8wpt compared with THC, respectively. This was also true of YFP+ cells that were co-labeled with either marker or both (Fig. 2A, C, E–E”’, I–I”’, T and Supplementary Fig. 2C). SVZ-RMS neurogenesis, however, was greatly diminished in TKO brains at 16wpt compared with the other two genotypes (Fig. 2J–L, M–R”’, S, T). DKO and THC brains had comparable levels of neurogenesis at 4wpt and 8wpt, despite scoring more Hoechst+ and YFP+ cells in the rRMS of DKO at 8wpt. While this result is possibly associated with a correction of the TKO phenotype by a single functional Rb allele, it is rather due to the fact that tamoxifen treatments were performed by IP injections in DKO mice only, which showed lower Cre recombination compared with gavage overall. Indeed, we detected significantly more progenitors and neuroblasts in both the aSVZ and rRMS in DKO versus THC at 16wpt (both treated by gavage) (Fig. 2J, K, M–P''', S, T and Supplementary Fig. 2A, C).

This data indicate that the Rb family negatively regulates NSCs self-renewal and is required for the proper control of the neuronal lineage development. Remarkably, a single Rb allele largely compensates for the dual loss of p130 and p107 and maintains a steady level of neurogenesis.

### A single Rb allele is sufficient to sustain neuronal maturation and survival in the OB in the absence of p107 and p130

**Fig. 3:**
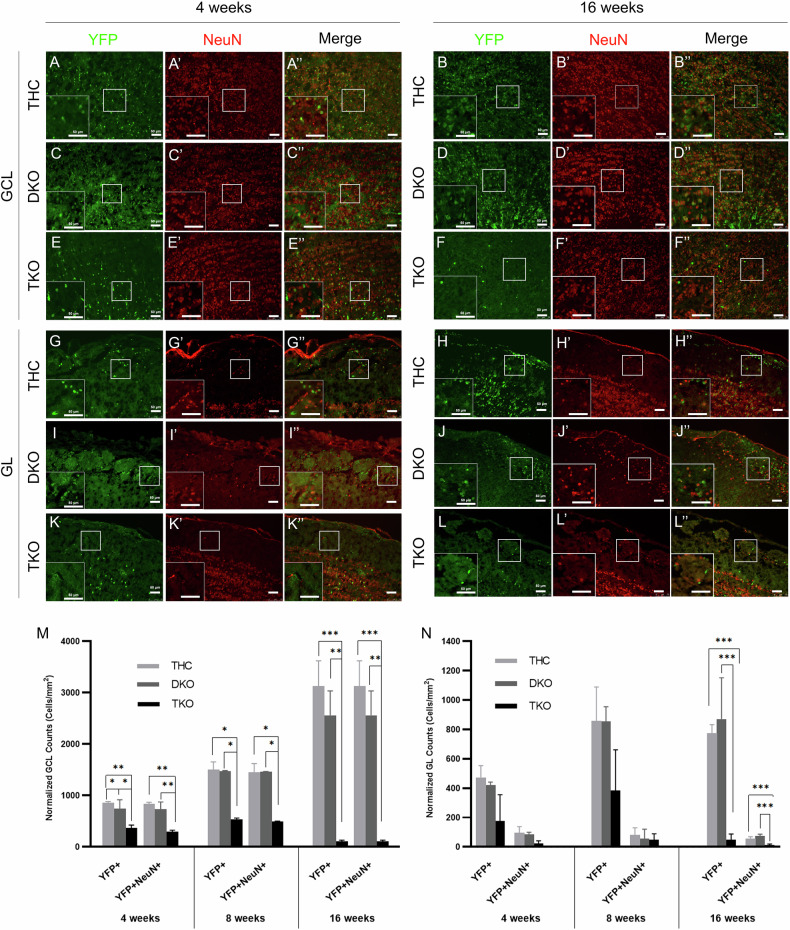
OB neurogenesis is lost in TKO mice by 16wpt but is preserved in DKO mice. IHC in the granule cell layer -GCL- (**A**–**F”**) and the periglomerular layer -GL- (**G**–**L”**) showing YFP+ and NeuN+ newborn neurons inside the OB at 4wpt and 16wpt across genotypes. Insets in panels show higher magnification images of the boxed regions in each layer. Quantification of cell counts inside the GCL (**M**) and GL (**N**) showing a steady accumulation of newborn neurons inside the OB layers in THC and DKO mice compared with a sharp decline in the level of neurogenesis in TKO brains between 4wpt and 16wpt. Scale bars, 50 µm. Error bars, mean ± SD. Unpaired 2-tailed Student’s t-test; *p < 0.05, **p < 0.01, ***p < 0.001. n = 3 biological replicates.

To assess OB neurogenesis in TKO mice, we quantified the numbers of YFP+ cells and [YFP + NeuN+] newborn neurons. Our results showed similar profiles of neurogenesis in THC and DKO brains that are characterized by a steady build-up of newborn YFP+ neurons up till 16wpt. Hence, in the GCL, YFP+ cells ranged between 744–856 cells/mm^2^ at 4wpt, 1479–1503 cells/mm^2^ at 8wpt, and 2561–3129 cells/mm^2^ at 16wpt, out of which 96-99% co-expressed the terminal differentiation marker, NeuN. In contrast, OB neurogenesis in TKO brains was sharply reduced by 46% at 4wpt and 96% at 16wpt compared with the other genotypes (Fig. 3A–F”, M). Similar findings were obtained in the GL, where an average of ~450 and ~850 YFP+ cells/mm^2^ are found in THC and DKO brains at 4wpt and 16wpt, compared with ~176 and ~49 cells/mm^2^ in TKO brains, thus corresponding to a reduction of 61% and 94%, respectively. Unlike in the GCL, only 7–20% of the YFP+ cells co-expressed NeuN in the GL in all three genotypes and across all time points (Fig. 3G–L”, N).

**Fig. 4:**
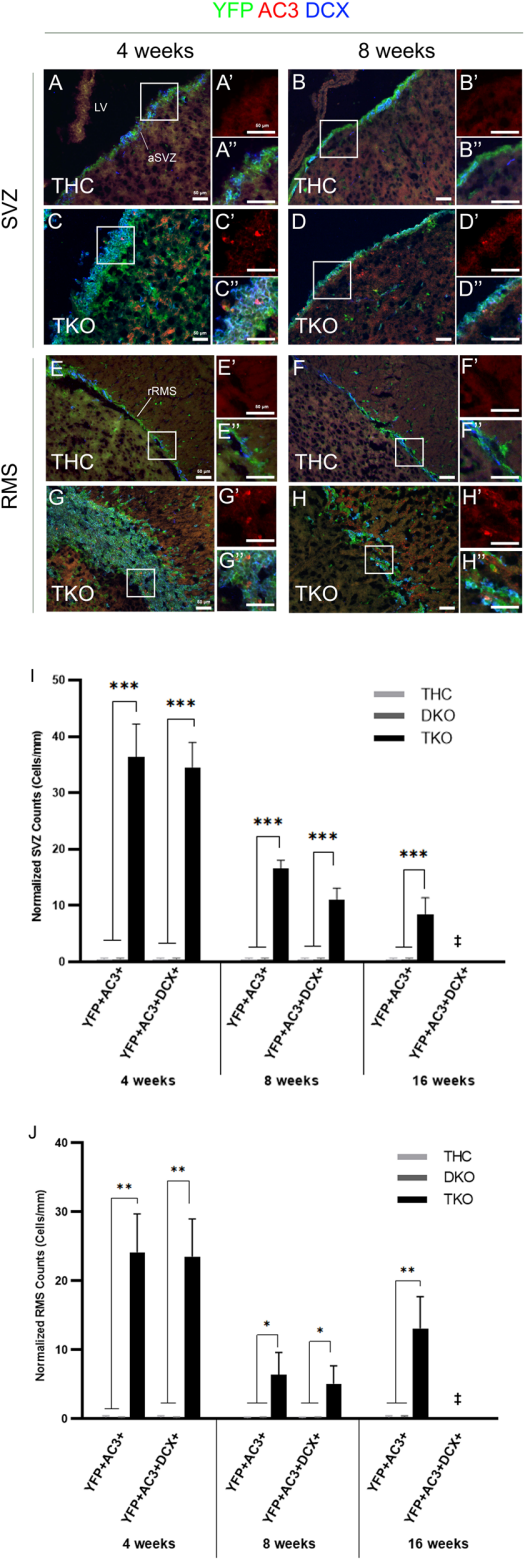
Loss of OB neurogenesis is due to severe survival defects in TKO mice. IHC on brain sagittal sections in the aSVZ (**A**–**D”**) and rRMS (**E**–**H”**) in THC versus TKO mice. Insets show higher magnification images of the boxed regions shown in each region (red; AC-3 staining, and triple staining). Note the massive cell death detected in TKO brains compared with THC at 4wpt and 8wpt. Quantification of cell counts inside of the aSVZ (**I**) and rRMS (**J**). TKO mice exhibit extensive cell death in both regions compared with THC and DKO mice. The majority of (YFP + AC-3+) cells co-express DCX in TKO mice, highlighting massive death in neuroblasts. ‡, Triple cell counts were not performed at 16wpt. Scale bars, 50 µm. Error bars, mean ± SD. Unpaired 2-tailed Student’s t-test; *p < 0.05, **p < 0.01, ***p < 0.001. n = 3–4 biological replicates.

To assess whether the dramatic decline in AN in TKO brains is due to survival defects, we co-immunostained for active caspase-3 (AC-3), YFP, and DCX. We indeed identified widespread cell death in the aSVZ and rRMS, where ~1.9–2.3% of the total YFP populations were AC-3-positive in TKO mice. Moreover, 70–95% of the apoptotic cells co-expressed DCX at 4wpt and 8wpt, indicating that these neuroblasts die before completing their terminal differentiation and reaching the OB (Fig. 4).

Our results demonstrate that AN in TKO brains is severely affected and lost by 16wpt due to major terminal differentiation and survival defects. Importantly, a single Rb allele can rescue this phenotype.

### Severe defects affecting NSCs’ lineage development upon loss of pocket proteins in the telencephalon

**Fig. 5:**
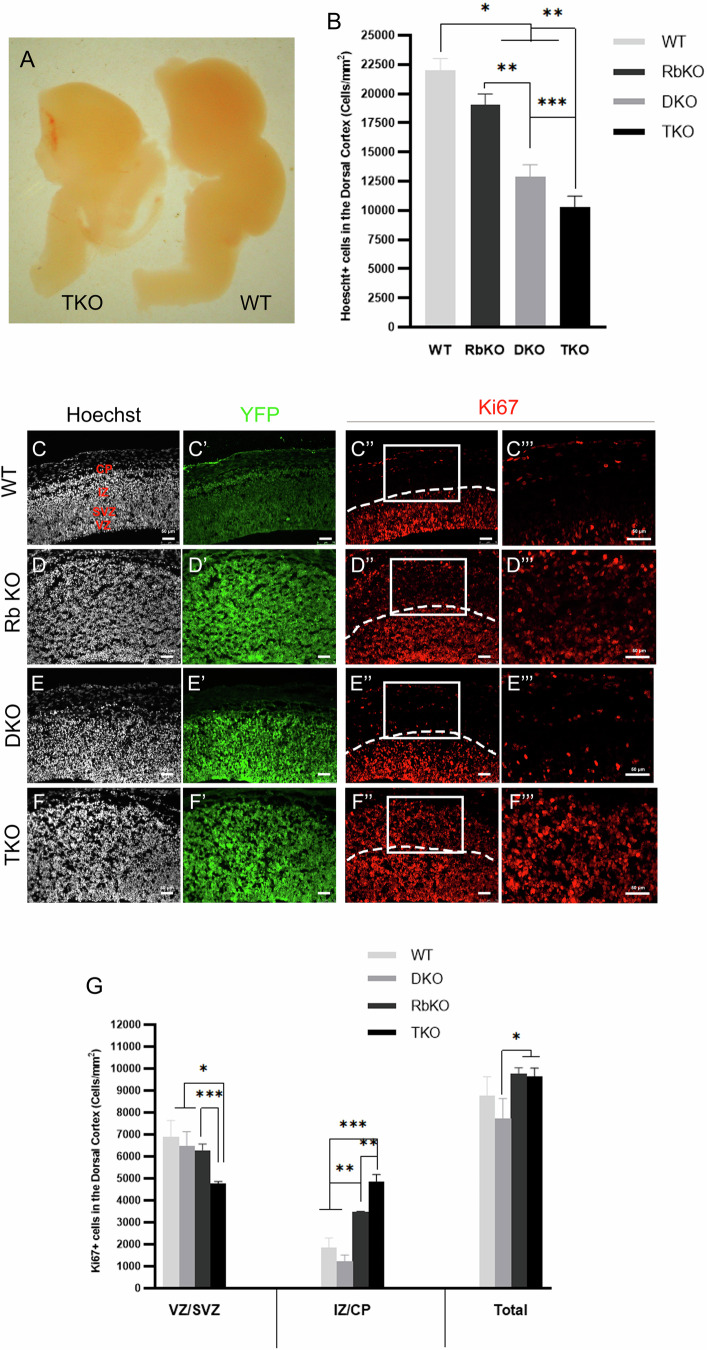
TKO embryos display a sharp reduction in brain size and exacerbated proliferation defects in the telencephalon. **A** Compared with WT embryos, TKO embryos display significant size reductions in the whole brain and the OBs, which are estimated to be around ~30% and ~85–90%, respectively. **B** Quantification of Hoechst-positive cells in the dorsal cortex (DC) at E14.5, showing the most prominent decrease in cell density in TKO embryos compared with the other genotypes. IHC on brain sagittal sections in the developing DC in WT (**C**–**C”’**), RbKO (**D**–**D”’**), DKO (**E**–**E”’**), and TKO (**F**–**F”’**) embryos. Insets in (**C”’**–**F”’**) are higher magnification images of boxed areas in (**C”**–**F”**). Note the ectopic proliferation in TKO and RbKO embryos inside the intermediate zone (IZ) and cortical plate (CP) (Ki67+ cells in red) compared with THC and DKO. VZ; ventricular zone, SVZ; subventricular zone. **G** Quantification of cell counts inside of the different cortical layers of the DC. Scale bars, 50 µm. Error bars, mean ± SD. Unpaired 2-tailed Student’s t-test; *p < 0.05, **p < 0.01, ***p < 0.001. n = 3–4 biological replicates.

Using the same mouse lines, we generated TKO, DKO, and RbKO embryos by treating pregnant females at E10.5, and conducted phenotypic analyses in comparison with WT embryos at E14.5. TKO embryos die at E17.5, while DKO and RbKO embryos die around birth. THC embryos revealed high variability in phenotype and, therefore, were excluded from this study. Successful Cre recombination and protein deletion(s) are evidenced by the uniform YFP expression throughout the telencephalon and the Western blot analyses, respectively (Fig. 5C’–F’ and Supplementary Fig. 1A).

Compared with WT embryos, TKO embryos had a remarkable reduction in brain size including the OBs (Fig. 5A). This was confirmed by the significant decrease in Hoechst cell density inside the dorsal cortex (DC) in TKO embryos by 53%, and DKO by 32% and RbKO (13.5%) embryos compared with WT (WT: 22032 ± 999 cells/mm^2^ vs TKO 10291 ± 939 vs DKO 12867 ± 1069 cells/mm^2^ vs RbKO 19061 ± 919) (Fig. 5B). Moreover, TKO embryos displayed severe cortical lamination defects (layer disorganization) inside the DC (Fig. 5F vs Fig. 5C). Next, we examined the various stages of NSCs’ lineage development. Co-immunostaining for YFP and Ki67 revealed that, in both WT and DKO embryos, 80–85% of all Ki67-positive cells were found in the VZ and SVZ, while the rest are located inside the IZ. In contrast, there was significant ectopic proliferation outside the proliferative zones in TKO and RbKO embryos; hence, 50.5% and 35.5% of Ki67+ cells were found scattered throughout the IZ and CP, respectively (Fig. 5C”–G). We identified similar proliferative defects in the basal ganglia in TKO and RbKO compared with DKO and WT (data not shown).

**Fig. 6:**
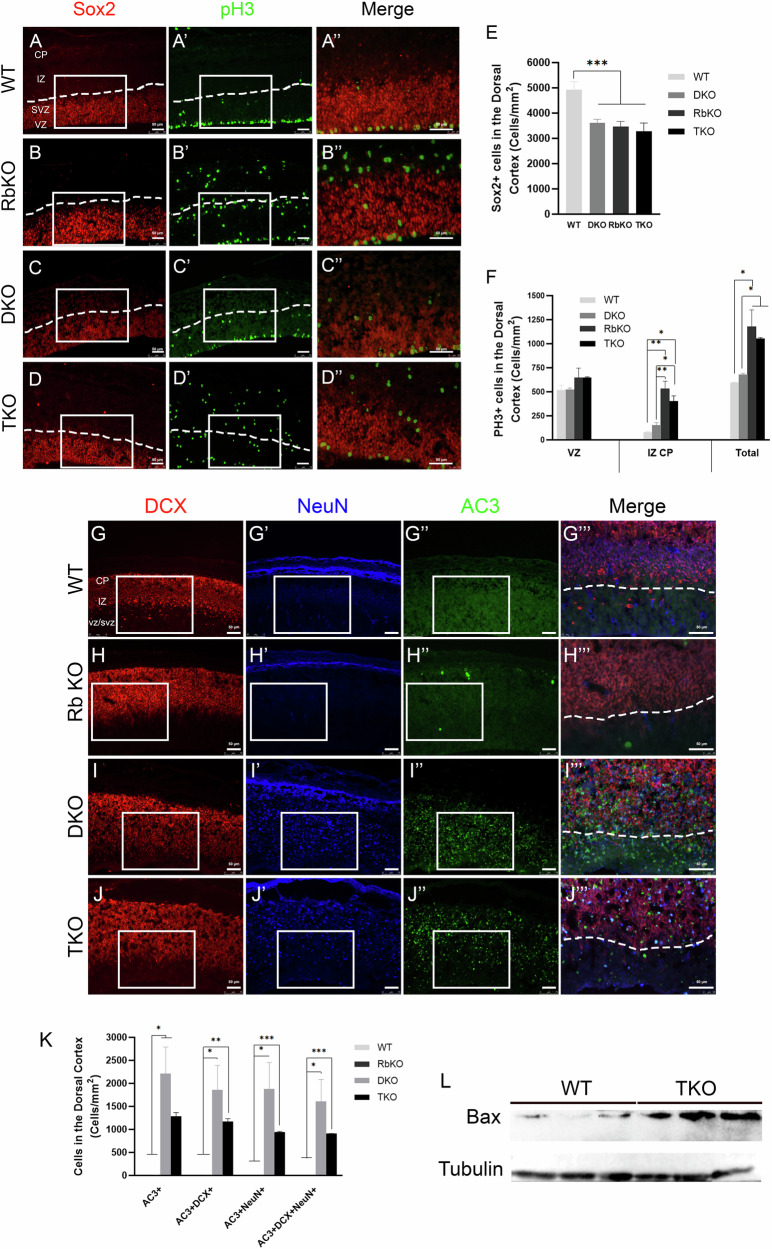
DKO embryos rescue the cell cycle exit defects but not the premature differentiation and survival defects observed in TKO embryos. IHC on sagittal brain sections in WT (**A**–**A”**), RbKO (**B**–**B”**), DKO (**C**–**C”**), TKO (**D**–**D”**) embryos at E14.5. **A”**–**D”** Higher magnification images of boxed areas in (**A**–**D’**). There is delayed cell cycle exit in RbKO and TKO embryos, as marked by several scattered PH3+ cells inside the IZ and CP compared with DKO and THC embryos. Dotted lines in **A**–**D**' mark the border between the SVZ and the IZ. Quantification of Sox2+ cells (**E**) and PH3+ cells (**F**) in the DC at E14.5. Note the significant decrease in Sox2+ population in all genotypes compared with WT embryos. **G**–**J”’** IHC in the DC showing DCX expression that is clearly confined to the IZ and CP in WT (**G**) and RbKO (**H**) embryos, but extended into the SVZ in DKO (**I**) and TKO (**J**) embryos. The latter two genotypes also show strong NeuN expression that is largely overlapping with DCX and AC-3, indicating severe differentiation and survival defects. Dotted lines in **G**'''–**J**''' mark the lower border of DCX expression in each genotype. **K** Quantification of cell counts in the DC at E14.5, confirming massive cell death of neuroblasts/neurons in DKO and TKO embryos. A very low number of or no AC-3+ cells were detected in WT and RbKO mice. **L** Western blot analysis showing 3.9-fold increase in Bax protein expression in TKO versus WT embryos at E14.5. Scale bars, 50 µm. Error bars, mean ± SD. Unpaired 2-tailed Student’s t-test; *p < 0.05, **p < 0.01, ***p < 0.001. n = 3–5 biological replicates.

Next, we assessed whether neuronal progenitors exit the cell cycle properly and migrate towards the cortical plate by co-immunostaining for Sox2 and PH3. Sox2 expression was confined to the VZ and SVZ in all genotypes; however, we scored a significant reduction in the size of this population in TKO, RbKO, and DKO by 33%, 29% and 26% compared with WT, respectively [cells/mm^2^: WT 4926 ± 324 vs TKO 3290 ± 322 vs RbKO 3473 ± 205 vs DKO 3618 ± 138, Fig. 6A–D”, E]. Also, we detected a 1.75- to 2-fold increase in the total number of PH3+ cells in TKO and RbKO but not in DKO compared with WT (cells/mm^2^: WT 596 ± 5 vs DKO: 680 ± 13 vs RbKO 1182 ± 171 vs TKO 1054 ± 10). Moreover, 55% and 62% of PH3+ cells were ectopically scattered throughout the whole DC in TKO and RbKO embryos, whereas the majority of PH3+ cells were mainly lining the VZ and IZ in DKO and WT embryos (Fig. 6A’–D”, F). These findings highlight the presence of ectopic proliferation and delayed cell cycle exit in the absence of all pocket proteins, as seen with single RbKO, a phenotype mainly driven by the loss of Rb.

To examine whether loss of pocket proteins affects neuronal differentiation and survival, we co-stained for DCX, NeuN, and AC-3. DCX expression strictly extended between the IZ and CP in WT and RbKO embryos; however, in DKO and TKO embryos, it was also detected in the SVZ, suggesting an earlier onset of neuronal differentiation (Fig. 6G–J). NeuN was not expressed in WT or RbKO embryos at this age, yet it showed strong and widespread expression in the SVZ, IZ, and CP as well as in the ganglionic eminences in DKO and TKO brains (Fig. 6G’–J’ and data not shown). The majority of NeuN-positive cells co-expressed DCX and AC-3, reflecting the presence of severe neuronal differentiation and survival defects in the whole telencephalon (Fig. 6G”–J”’, K). We detected a 3.9-fold increase in Bax expression in TKO versus WT embryos, consistent with the widespread apoptosis (Fig. 6L).

Taken together, our data demonstrate that loss of all pocket proteins severely disrupts all stages of neuronal lineage development. Notably, a single wt Rb allele is sufficient to rescue the proliferation and cell cycle exit defects but not the differentiation and survival defects. To assess whether p107 or p130 can in turn rescue the latter defects, we examined the phenotypes of (Rb−/−; p130−/−; p107+/−) and (Rb−/−; p130+/−; p107−/−) carrying one wild-type allele of each gene, respectively. Our results showed that both DKOs recapitulated the same differentiation and survival defects observed in the DKO carrying a single functional Rb allele (in addition to the proliferation defects associated with loss of Rb) (Supplementary Fig. 3A–C”’, E–G”’, I, J, K). In contrast, embryos carrying two functional alleles, e.g., Rb−/−; p130+/−; p107+/−, did not show the above defects (data not shown). This data indicates that pocket proteins play interchangeable roles in a dose-dependent manner in the control of neuronal differentiation and survival during development.

### Distinct deregulations in the Notch-Hes signaling pathway in the embryonic versus adult brain in TKO mice/embryos

**Fig. 7:**
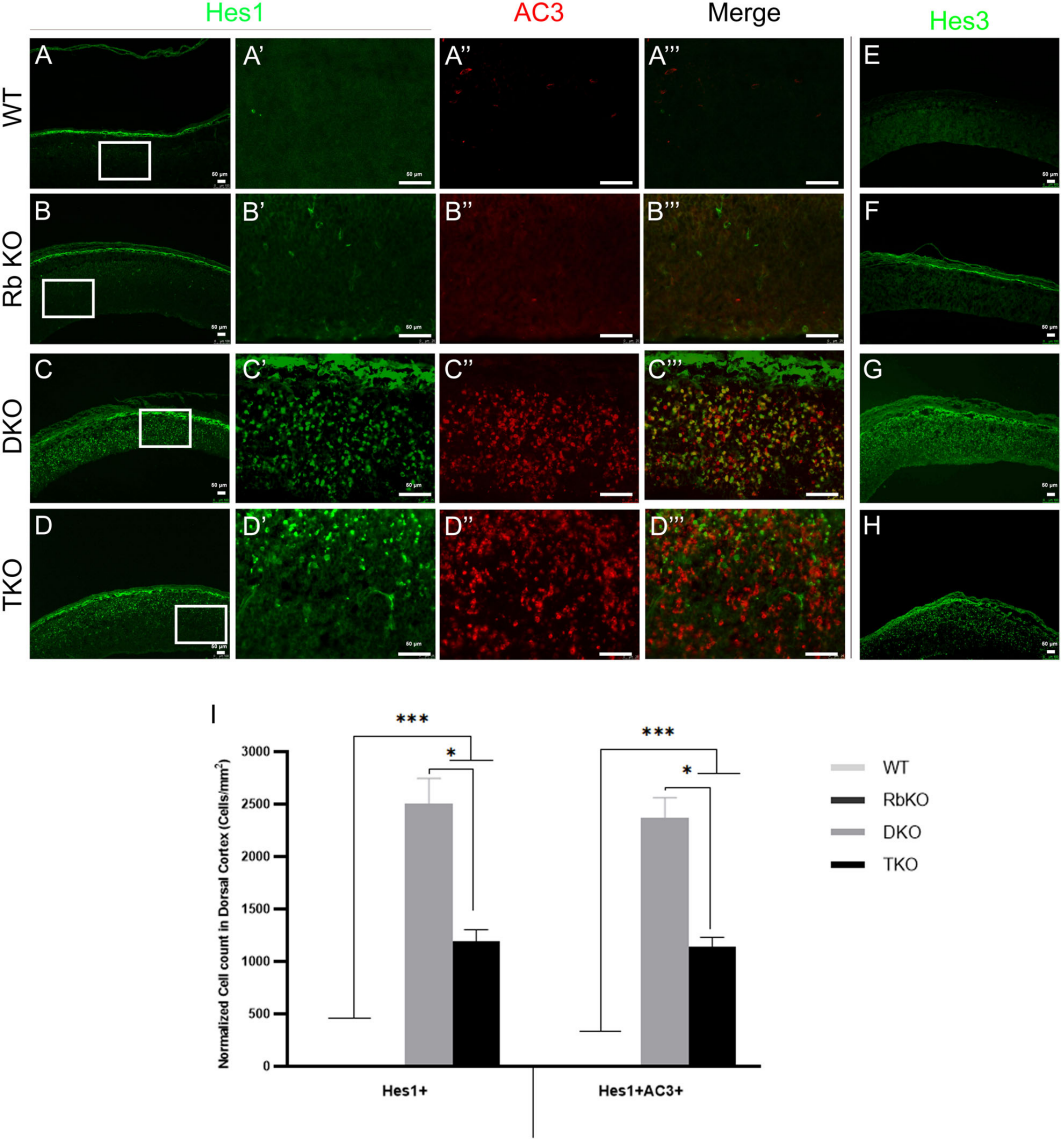
Abnormal Hes1 and Hes3 expressions in the telencephalon in DKO and TKO embryos. IHC on brain sagittal sections in WT (**A**–**A”’**, **E**), RbKO (**B**–**B”’**, **F**), DKO (**C**–**C”’**, **G**), and TKO (**D**–**D”’**, **H**) embryos showing Hes1 and Hes3 staining throughout the DC in the latter two genotypes only. **A'**–**D”’** Higher magnification images of boxed areas shown in (**A**–**D**). The majority of Hes-positive cells co-express AC-3 (but not Ki67; data not shown). **I** Quantification of cell counts inside the DC at E14.5. A very low number of or no Hes1+ cells were detected in WT and RbKO mice. Scale bars, 50 µm. Error bars, mean ± SD. Unpaired 2-tailed Student’s t-test; *p < 0.05, **p < 0.01, ***p < 0.001. n = 3 biological replicates.

**Fig. 8:**
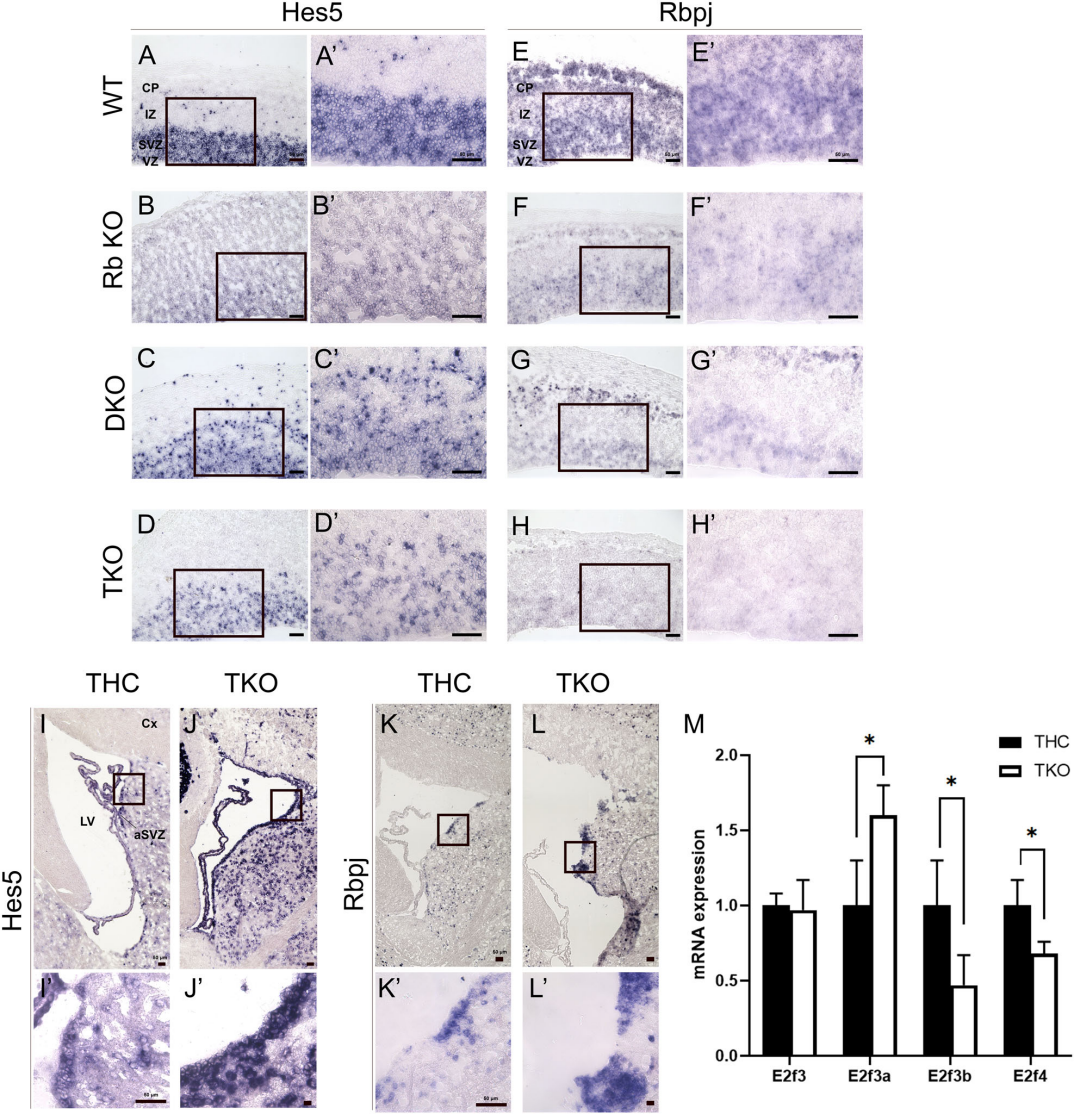
Opposed deregulations in *Hes5* and *Rbpj* transcript levels in the embryonic versus adult brain upon loss of pocket proteins. In situ hybridization with antisense probes for *Hes5* (**A**–**D’, I**–**J’**) and *Rbpj* (**E**–**H’, K**–**L’**) performed on embryonic (**A**–**H’**; E14.5) and adult (**I**–**L’**; 4wpt) brain sagittal sections. **A’**–**L’** are higher magnification images of black boxes shown in (**A**–**L**). *Hes5* and *Rbpj* mRNA transcript levels are significantly downregulated in the VZ-SVZ in TKO, RbKO, and DKO compared with WT embryos during development. The levels of both transcripts are, however, strongly upregulated in the aSVZ, Cx, and striatum in TKO mice compared with THC mice. **M** qRT-PCR results performed on cDNA derived from embryonic forebrain of TKO (n = 3) versus WT (n = 4) embryos at E14.5 and showing downregulated E2F3b and E2f4 expressions as opposed to upregulated E2F3a expression. Data is normalized to the 18S gene expression at internal. Legend as in Figs. 1 and 5. Scale bars, 50 µm. n = 3–4 biological replicates.

Given that Notch signaling plays a key developmental role in maintaining the proper balance between cell proliferation and the timing of differentiation, we investigated whether a potential deregulation in this pathway could be mediating the observed defects. We thus assessed the expressions of *Notch2*, *Hes1*, *Hes3*, *Hes5*, and *Rbpj* in the developing and adult brain. While we did not detect any Hes1 or Hes3 protein expression in WT and RbKO embryos or the adult brain, both proteins were strongly expressed throughout the dorsal and ventral cortices in all three combinations of DKO embryos and TKO embryos (Fig. 7A–D’, E–H, Supplementary Fig. 3D, D’, H, H’, and data not shown). Notably, only 8–14% of Hes1+ cells (and Hes3+) co-expressed Ki67 while the majority stained positive for both DCX and AC-3, indicating that they are postmitotic neuroblasts undergoing apoptosis (Fig. 7A”–D”’, I, Supplementary Fig. 3D”, D”’, H”, H”’, and data not shown). In contrast, compared with WT, we detected significant downregulation in the transcript levels of *Notch2, Hes5,* and *Rbpj* inside the VZ-SVZ in TKO embryos as well as RbKO and DKO, albeit to a less extent (Fig. 8A–H’ and data not shown). In comparison, *Hes5* and *Rbpj* mRNA levels were strongly upregulated in the aSVZ and rRMS as well as in scattered cells in the striatum and the dorsal cortex in TKO brains compared with THC (Fig. 8I–L’). Given that Notch1, Hes1/5, and Rbpj are common transcriptional targets of E2f3, specifically E2F3b, and to a less extent E2F4 [28], we assessed the transcript levels of these E2Fs in TKO versus WT embryos at E14.5. Our qRT-PCR results in TKOs indeed showed 53% and 32% reductions in E2F3b and E2F4 mRNA expression levels, respectively, as opposed to 60% increase in E2F3a expression, and no significant change in the total E2F3 level (Fig. 8M). Interestingly, such imbalance in E2F3a/b expressions could well justify the decrease in Sox2 expression and the premature differentiation defects observed earlier (refer to discussion and [29]). Altogether, the above findings highlight opposed deregulations in the Notch-Hes pathway, which could be mediating the distinct phenotypes observed in the adult versus embryonic brain.

## Discussion

Here, we identify Rb as a critical pocket protein in maintaining adult neurogenesis in the aSVZ-OB. Previous reports have also shown that Rb plays the most vital role in cancer suppression in the CNS, including the adult retina [5, 7]. However, during development, Rb fails to rescue the severe differentiation and survival defects that are associated with compound loss of p107 and p130. Notably, in this paper, the same defects are detected when any five out of the six alleles of pocket proteins are lost (but not four alleles), suggesting the presence of a dose-dependent requirement for these proteins with interchangeable roles among them during NSC lineage development.

### Expansion of Rb’s involvement during adult neurogenesis to maintain all neurogenic stages

Several reports have described the role of Rb in the regulation of developmental and adult neurogenesis by mainly relying on single KO models [14–16, 22, 30, 31]. Compensation by Rb family members was therefore a reasonable explanation for the missing or at least partial involvement of Rb in any neurogenic stage, as seen with lineage development of Rb-deficient cortical progenitors [14, 15] and aSVZ-OB neurogenesis following Rb inducible deletion [16]. Here, we accounted for the compensatory background by comparing DKO with TKO mice, whereby a single functional Rb allele was sufficient to prevent a severe phenotype of aNSCs activation and depletion. Our earlier study using Nestin-CreER^T2^ inducible RbKO model only implicated Rb in regulating SVZ progenitors’ proliferation and long-term survival of adult-born OB neurons [16]. It is now possible to conclude that Rb is indeed active along the molecular pathways of all adult neurogenic processes. However, this does not exclude the possibility of long-term defects unfolding in older DKO mice (after 16wpt). On the other hand, it remains to be determined whether a single functional allele of p107 or p130 can also rescue the adult TKO phenotype. In fact, when compared with THC, DKO mice carrying two functional p107 alleles (Rb−/−; p130−/−; p107+/+) display similar level of neurogenesis in the aSVZ and RMS at 16wpt, however; the number of newborn neurons is reduced by half inside the OB (Supplementary Fig. 4). This data and the above hypothesis require further investigation in the future.

During embryonic neurogenesis, given the striking similarities in phenotypes among all three DKOs and TKO, there seem to be fewer exclusive roles attributed to Rb or any other pocket protein, which do not overlap with the functions of the two other family members. The only compensatory role ascribed to Rb is preventing ectopic proliferation and delayed cell cycle exit. Rb, however, could not reverse the severe differentiation and survival defects observed in the absence of p107 and p130, which are also detected after loss of any combination of five alleles of pocket proteins. Rb-p107 DKO embryos exhibited Bax-mediated cortical cell death at E17.5 [18], similar to what we reported in DKO and TKO embryos, suggesting that the absence of compensatory effects is not specific to Rb. Rather, cortical neurogenesis might dictate the involvement of at least two functional pocket proteins to prevent severe dysregulation of brain development. It might also be plausible that the increase in Rb’s functional redundancy from embryonic to adult neurogenesis (as to allow for more compensation by p107 and p130) is a developmental mechanism intended to sustain the adult neurogenic pool throughout life. This was indeed the case of another cell cycle regulator, p57, whose deletion prevents the emergence of adult NSCs [32]. Whether Rb acts in a similar manner awaits further robust proof.

### Involvement of Notch-Hes pathway in TKO neurogenic defects

Oshikawa et al. reported that, in a TKO of pocket proteins induced in cortical progenitors, neuronal differentiation was not affected with no signs of apoptosis [19], which contradicts our findings showing high percentage of apoptotic neuroblasts/neurons in the developing TKO cortex. This could be explained by the facts that in Oshikawa’s study: 1) gene deletion by *in utero* electroporation does not systematically target all stem and progenitor cells in the Nestin lineage and the concentration of electroporated plasmids gradually decreases with each division, and 2) TKO induction was performed at E14.5 and phenotypic analysis at P2 and P10, which could have underestimated the extent of apoptotic cell death at earlier time points [33].

In search for the molecular mediator(s) of the phenotypic defects observed upon loss of pocket proteins, we implicated opposed deregulations in the Notch-Hes pathway in the embryonic versus adult brain. While such disruptions could be secondary effects to the developmental defects, we strongly argue that they play direct role(s) based on several lines of evidence. First, the Notch pathway promotes astrocytic fate and directly represses oligodendrocyte development [34, 35]. We indeed report in TKO brains an upregulation in astrocytic lineage derived from NSCs in the aSVZ (Fig. 1H-K) and aSGZ [21]. Our previous transcriptomics analysis of aSVZ-NSCs in TKO brains uncovered significant downregulation in oligodendrocyte specification markers such as MBP, MYRF, OMG, MOBP, and PLP1, as well as RITA (Rbpj interacting and tubulin-associated 1 protein short), a direct modulator of Notch signaling that mediates nuclear export of Rbpj, hence decreasing its transcriptional activity ([21]: GSE190766, [36]). This is consistent with the upregulated transcript expressions of Rbpj and its direct target Hes5 in TKO brains.

Second, loss of pocket proteins is known to be associated with deregulated expressions of their target E2F genes, e.g., E2F1-5 [37]. Here, we show that the imbalance in E2F3a and E2F3b transcript levels is directly linked to the phenotypic defects reported in TKO embryos. Although E2F3b is classically considered a transcriptional repressor [38–40], Julian et al. showed that, in E2F3b−/− neurospheres, half of the direct E2F3b targets, including Sox2, Hes5, and Rbpj had reduced mRNA levels as opposed to increased expression in Hes1 [28]. This data is fully consistent with our findings (Figs. 6E, 7 and 8) and argues that E2f3b indeed can function (although not exclusively) as a transcriptional activator. Moreover, E2F3a and E2F3b play opposing roles in the regulation of Sox2 expression during NSC fate determination. Hence, in the absence of E2F3b, E2F3a-p107-mediated repression dominates and reduces Sox2 levels, thereby increasing neurogenesis at the expense of progenitor proliferation [29]. We believe that this mechanism, in addition to downregulation of Notch signaling, contributes to the premature differentiation defects and reduction in brain size observed in TKO embryos at least partially. This was also shown to be the case following inducible deletion of Rbpj in telencephalic NSCs that prematurely differentiated into neurons and were depleted [41]. Add to this is the decreased Sox2 expression, which was previously shown to be a direct target of Rbpj/N1ICD in cortical NSCs in vivo [42].

Third, we found that Bax expression, a downstream effector of the p53 pathway, is strongly upregulated in TKO embryos along with Hes1 and Hes3. The p53 pathway was found to trigger cell death in neural progenitor cells overexpressing Notch1 intracellular domain [43]. At E10.5, Hes3 expression is restricted to the isthmus, while Hes1 and Hes5 are widely expressed in the CNS, and their expressions become restricted to the VZ thereafter [44]. While the reason behind Hes3 upregulation is not clear (or whether it could be linked to cell death in neurons), Hes1 overexpression can be attributed to the loss of p107, its direct transcriptional repressor, as previously shown in p107−/− mice [11]. Alternatively, neuronal apoptosis during development might also be a secondary effect of stage disruptions such as abnormal overlap between precursor proliferation, commitment, and/or differentiation.

## Supplementary information

Supplementary Figures Legends

Supplemental Figure 1

Supplemental Figure 2

Supplemental Figure 3

Supplemental Figure 4

Supplementary Material—Original Western Blots

## Data Availability

All data generated or analyzed during this study are included in this published article and its supplementary information files.
