# Supplementary material for: Rb substantially compensates for the double loss of p130 and p107 in adult but not embryonic neural stem cell lineages
Source: Cell Death Dis. 2025 Jul 10;16(1):511. doi: 10.1038/s41419-025-07815-6 (PMC12246042; doi:10.1038/s41419-025-07815-6)
Supplement: Supplementary file 1 — Supplementary Figures Legends [file 41419_2025_7815_MOESM1_ESM.docx]

**Supplementary Figures Legends**

**Supplementary figure 1**

**(A)** Western blot analyses showing successful Cre recombination and pocket proteins’ deletions in TKO versus WT embryos at E14.5. **(B-D)** Quantification of cell counts in the aSVZ and rRMS showing no phenotypic differences between THC embryos and No Cre-THC controls at all time-points examined. n = 3 biological replicates at least.

**Supplementary figure 2**

**(A, C)** Quantification of single cell counts inside the aSVZ (A) and rRMS (C) in all four genotypes at all timepoints examined. **(B)** IHC on brain sagittal section in TKO mice at 4wpt showing lateral ventricle enlargement and ventricular heterotopia frequently detected along the ventral side of the aSVZ. Scale bars, 50 µm. Error bars, mean ± SD. Unpaired 2-tailed Student’s t test; *p < 0.05, **p < 0.01, ***p < 0.001. n = 3 biological replicates.

**Supplementary figure 3**

**(A-H’’’)** IHC staining on sagittal sections in the dorsal cortex (DC) of two DKO embryos: (Rb-/-; p107-/-;p130+/-) and (Rb-/-;p107+/-;p130-/-) at E14.5 showing similar developmental defects (premature neuronal differentiation and massive cell death) compared with the DKO (Rb+/-;p107-/-;p130-/-) described in figures 5-7. **(I-K)** Quantification of cell counts in the DC at E14.5 with the indicated markers. Scale bars, 50 µm. Error bars, mean ± SD. Unpaired 2-tailed Student’s t test; *p < 0.05, **p < 0.01, ***p < 0.001. n = 2-4 biological replicates.

**Supplementary figure 4**

**(A-C’’’)** Triple IHC staining on adult brain sagittal sections of (Rb-/-; p130-/-; p107+/+) mice at 16wpt showing a similar level of neurogenesis in the aSVZ and RMS compared with THC. Inset panels show higher magnification images of dashed boxes in the aSVZ and rRMS. **(E-F’’)** Double IHC staining inside the OB. **(D and G)** Quantification of cell counts in (A-F’’) compared with THC. Scale bars, 50 µm. Error bars, mean ± SD. Unpaired 2-tailed Student’s t test; *p < 0.05, **p < 0.01. n = 3 biological replicates.
