# Supplementary figures and images for: Rb substantially compensates for the double loss of p130 and p107 in adult but not embryonic neural stem cell lineages
Source: Cell Death Dis. 2025 Jul 10;16(1):511. doi: 10.1038/s41419-025-07815-6 (PMC12246042; doi:10.1038/s41419-025-07815-6)

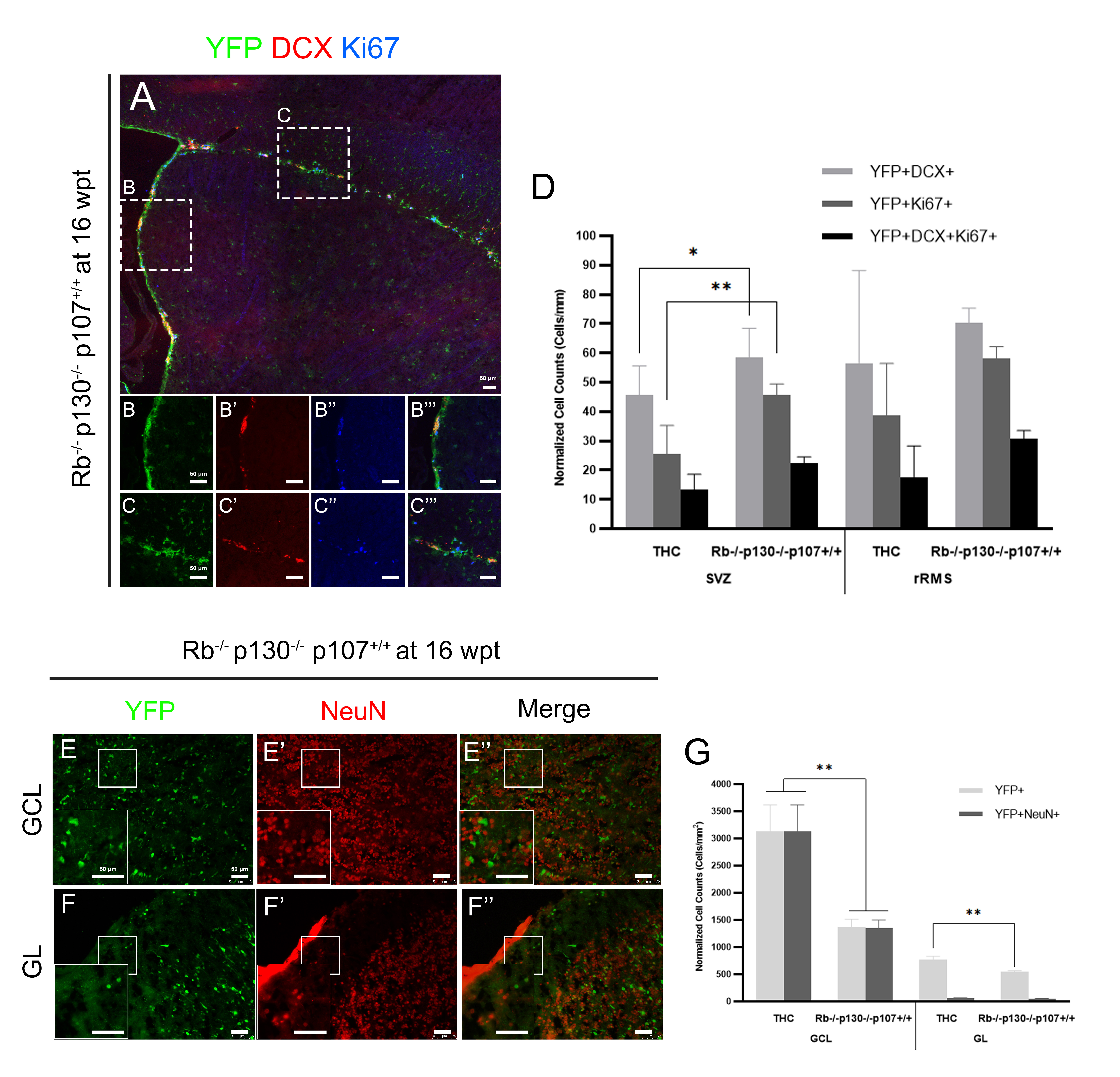

Supplement: Supplementary file 5 — Supplemental Figure 4 [file 41419_2025_7815_MOESM5_ESM.jpg]

Supplemental Material

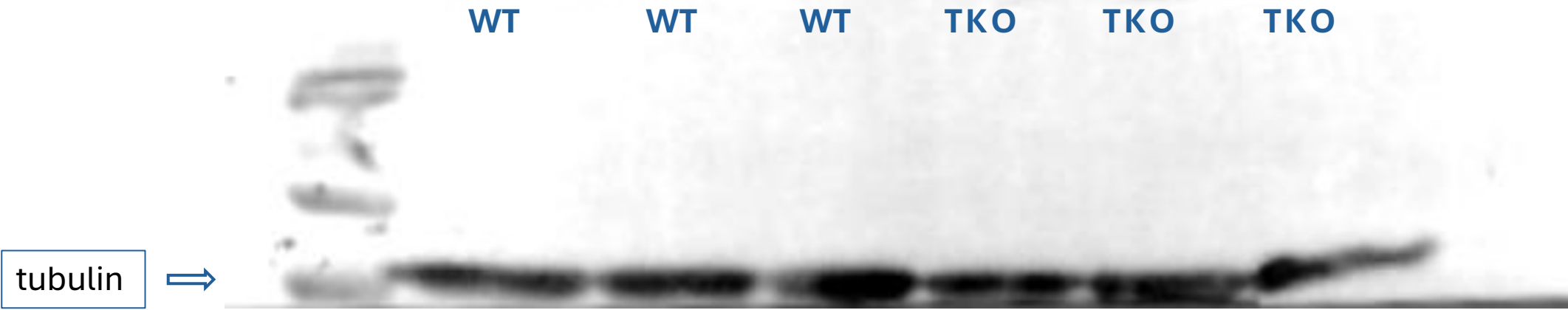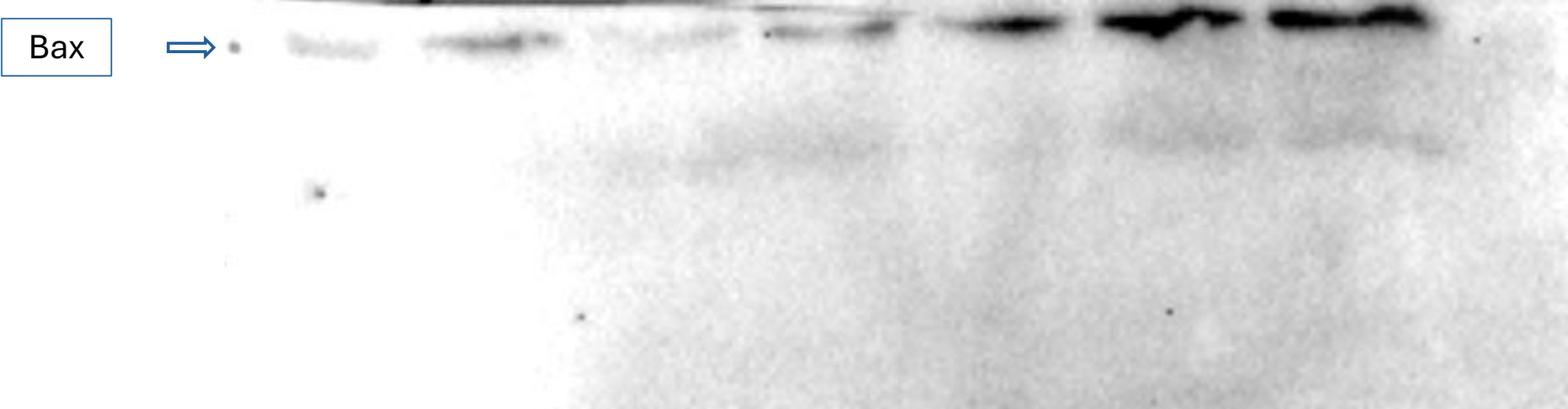

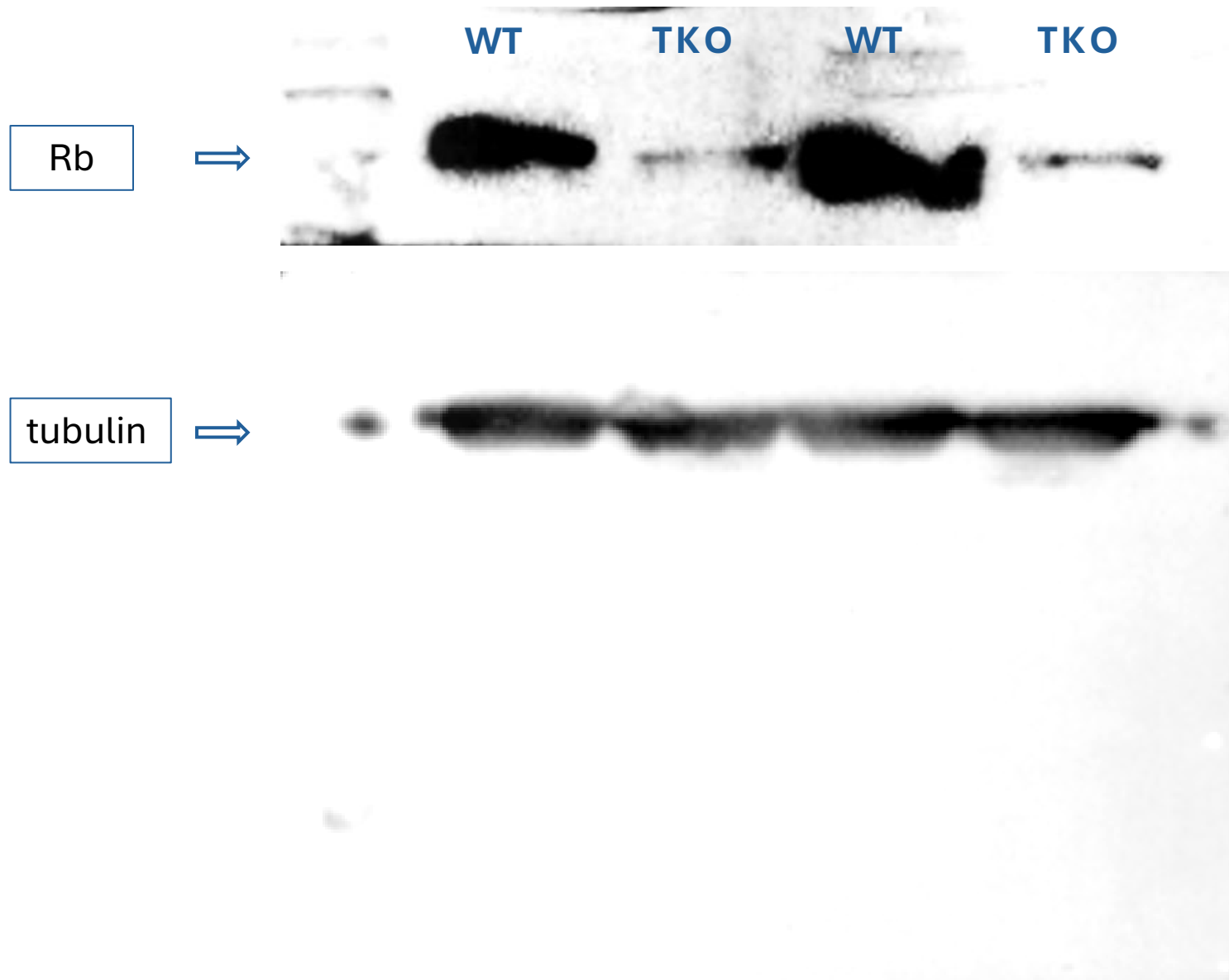

P107

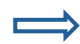

tubulin

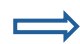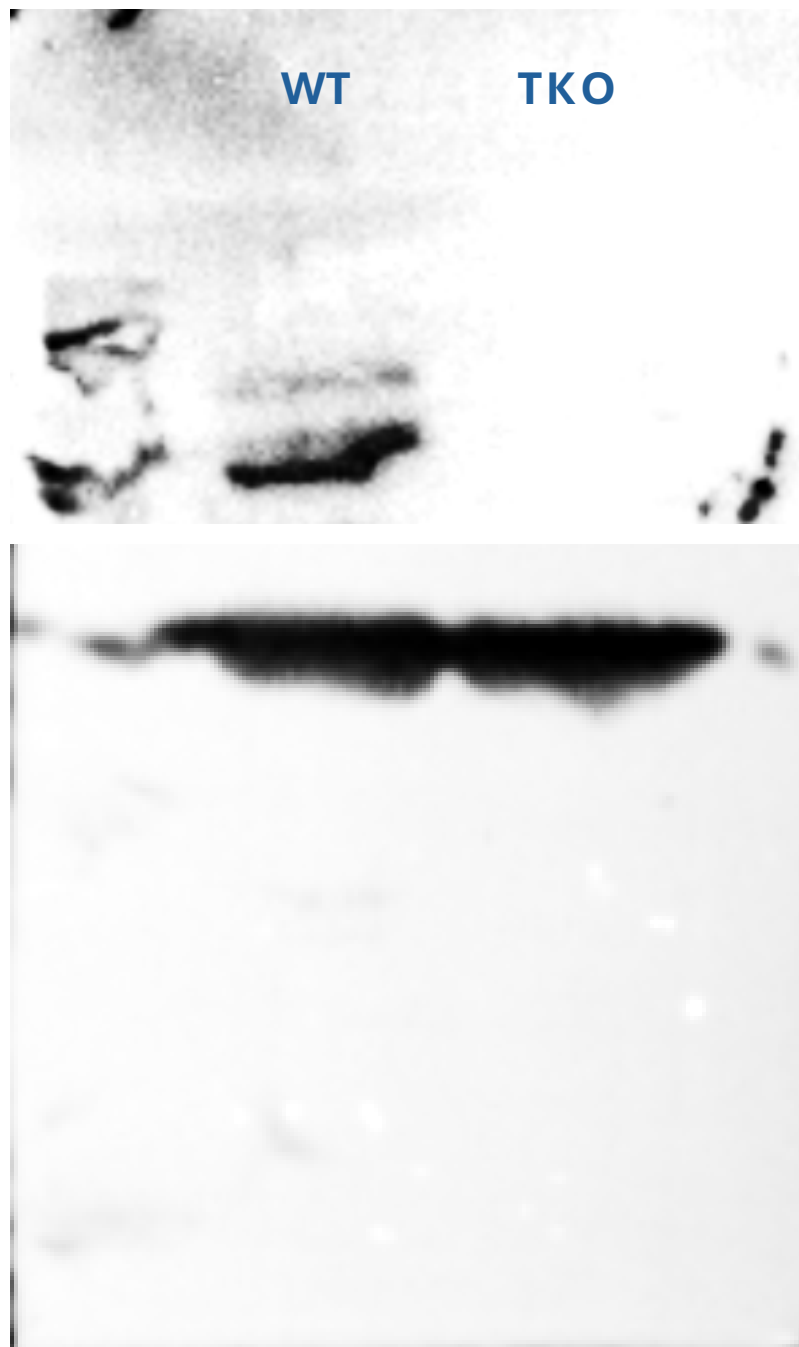

p130

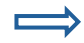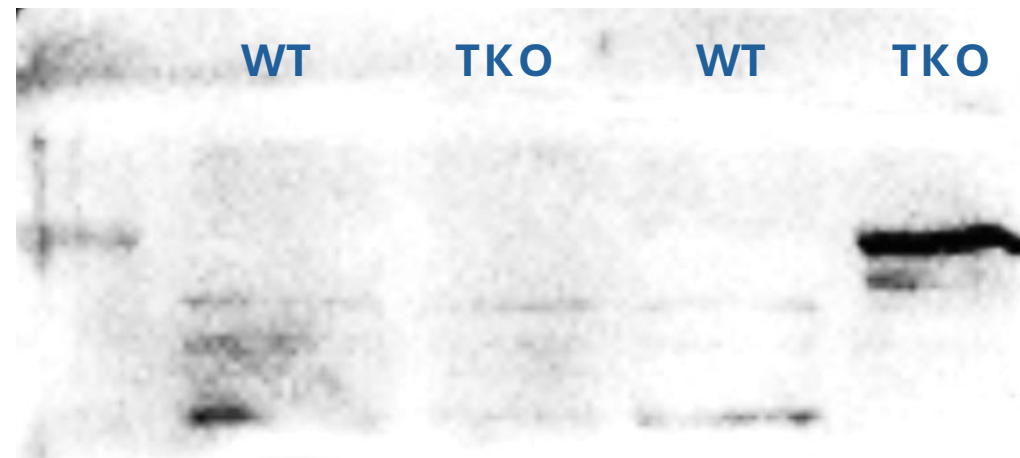

tubulin

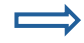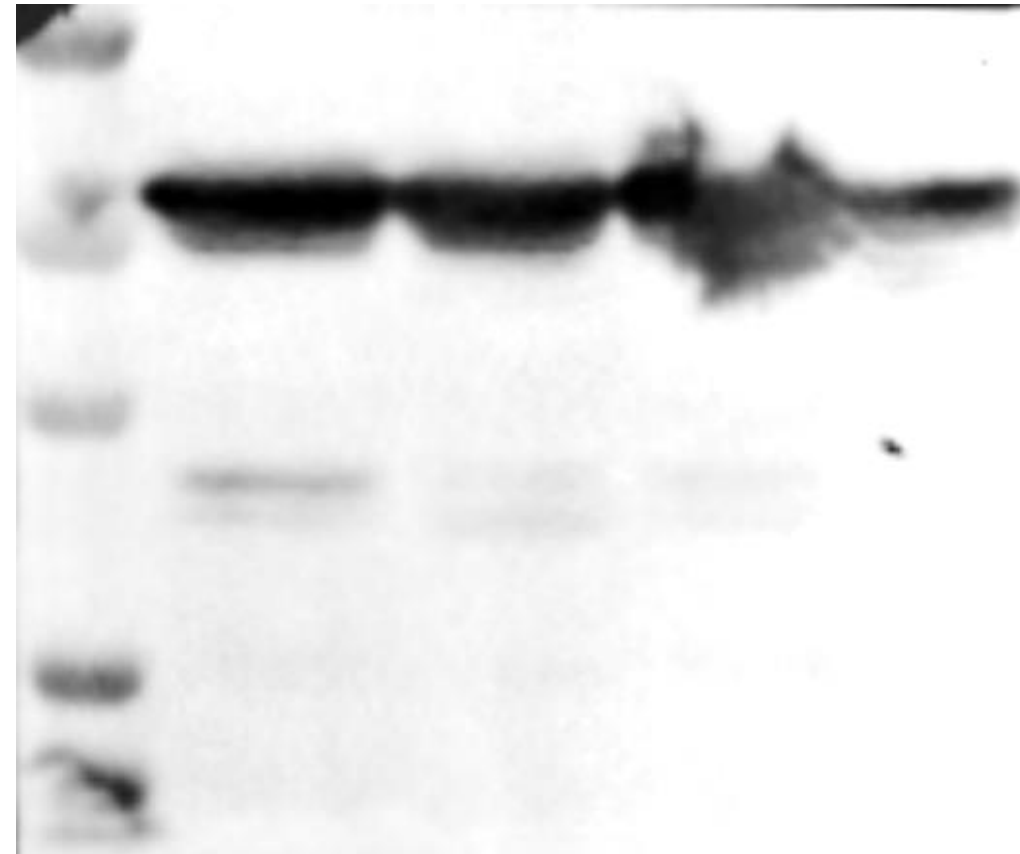

Supplement: Supplementary file 6 — Supplementary Material—Original Western Blots [file 41419_2025_7815_MOESM6_ESM.pdf]
